# Climate‐driven phenological shifts in emergence dates of British bees

**DOI:** 10.1002/ece3.10284

**Published:** 2023-07-09

**Authors:** Chris Wyver, Simon G. Potts, Mike Edwards, Rowan Edwards, Stuart Roberts, Deepa Senapathi

**Affiliations:** ^1^ Centre for Agri‐Environmental Research, School of Agriculture, Policy and Development University of Reading Reading UK; ^2^ Bees, Wasps and Ants Recording Society West Sussex UK

**Keywords:** bees, climate change, phenological shift, phenology

## Abstract

Climate change has a diverse range of impacts on wild bees, including their phenology or timing of life history events. Climate‐driven phenological shifts can not only impact individuals at species level but also threaten the vital pollination service that wild bees provide to both wild plants and cultivated crops. Despite their involvement in pollination, for most bee species, especially in Great Britain, little is known about phenological shifts. This study makes use of 40 years of presence‐only data for 88 species of wild bees to analyse shifts in emergence dates, both over time and in relation to temperature. The analyses reveal widespread advances in emergence dates of British wild bees, at an average rate of 0.40 ± 0.02 days per year since 1980 across all species in the study data set. Temperature is a key driver of this shift, with an average advance of 6.5 ± 0.2 days per 1°C warming. For change in emergence dates both over time and in relation to temperature, there was significant species‐specific variation, with 14 species showing significant advances over time and 67 showing significant advances in relation to temperature. Traits did not appear to explain variation in individual species' responses, with overwintering stage, lecty, emergence period and voltinism considered as possible explanatory traits. Pairwise comparisons showed no differences in sensitivity of emergence dates to increasing temperature between trait groups (groups of species which share all four traits) that differed by only one trait. These results highlight not only a direct impact of temperature on the phenology of wild bees themselves but also the species‐specific shifts highlight a possible impact on the temporal structure of bee communities and the pollination networks for which the wild bees are so crucial.

## Graphical abstract

This study uses 40 years of data to assess trends in emergence dates of 88 species of wild bee in Great Britain. Changes in emergence dates varied between species, although there was a general trend of earlier advancement in warmer years, indicating some level of phenological sensitivity to climate change. These advances were poorly explained by life‐history traits.

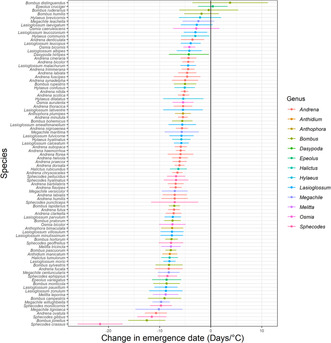

## 1 INTRODUCTION

Many taxa, including plants (Büntgen et al., 2022), birds (Crick & Sparks, 1999) and insects (Hassall et al., 2017) have been shown to shift their phenologies in response to the changing climate. For example, in the UK, the first flowering dates of plants are advancing by an average of 5.4 days per decade (Büntgen et al., 2022), and the emergence dates of hoverflies are advancing at an average rate of 12.5 days per 1°C temperature increase (Hassall et al., 2017). Wild bees in the US have also been shown to be impacted, with evidence of climate‐driven phenological shifts in emergence dates of 10.4 days over the last 130 years (Bartomeus et al., 2011) and 4.3 days per year (Dorian et al., 2022). While it might seem sensible to predict widespread advances in phenology under warmer conditions through increased rates of metabolic processes, these mechanisms and the impact of climate change on them remain poorly quantified (Fründ et al., 2013). Advances are also not guaranteed, with warmer temperatures linked to slower completion of the prepupal stage in *Osmia bicornis* (Radmacher & Strohm, 2011). In either case, the impact of climate on phenological shifts in bee emergence for many species, particularly in Great Britain, is yet to be studied in depth.

Phenological shifts can impact not only the individual species but also the ecosystem services they provide such as pollination. Globally, animals, including wild bees contribute significantly to the pollination of plants. This includes up to 87.5% of angiosperms (Ollerton et al., 2011) and around 75% of cultivated crop species (Klein et al., 2007). Great Britain is home to some 270 species of wild bees, and they increase agricultural productivity in the region by an estimated £630 million (Breeze et al., 2021). The list of crops where yield and/or quality is improved when insect pollination occurs includes many fruits and vegetables such as apples (Garratt et al., 2014) and pears (Fountain et al., 2019), both of which are commonly cultivated in Great Britain.

Phenological shifts could have mixed repercussions for bee fitness. Earlier emergence, for example, could benefit a particular species, by reducing competition for forage should the phenologies of competitors not keep pace. The same species may benefit more generally from an elongated growing season. Conversely, the same species could also experience an increased risk of exposure to suboptimal temperatures (Iler et al., 2021) both directly and indirectly, through damage to the plants on which they forage.

Bee species exhibit a wide range of life‐history strategies, which influence the timing of emergence dates (Stemkovski et al., 2020). These strategies may also influence their phenological sensitivity to climate change, as shown in solitary bees in the US (Dorian et al., 2022) and Canadian butterflies (Kharouba et al., 2014). There are a range of traits that could influence the sensitivity of emergence dates to climate change. For example, butterflies that overwinter as adults tend to advance their phenologies more than those butterflies that overwinter as larvae or pupae (Diamond et al., 2011). This is possibly because by overwintering as adults, these butterflies can respond to favourable temperatures without further development (Dennis, 1993), in comparison to species that overwinter as larvae or pupae, which require additional time to reach maturity. More generally, there is also evidence from solitary bees to suggest that early emerging species' phenology is more sensitive to climate change, compared with those that emerge later in the year (Dorian et al., 2022).

Other life history traits may also play a role in determining bee emergence. Lecty, for example, may impact sensitivity. Lecty ‘determines the breadth of resources that a bee exploits: oligolectic bees collect pollen from a narrow range of (usually related) plant genera, while polylectic bees have a broader diet’ (Ogilvie & Forrest, 2017). Oligolectic species must remain in temporal synchrony with the plants they forage on, which in the case of some species is restricted to plants that only flower for a short duration. These oligolectic bees could have different phenological sensitivities to climate change than polylectic species (Minckley et al., 2013). Polylectic species are adapted to forage on a range of plants, and are therefore under less pressure to track the flowering dates of a particular plant or group of plants.

Alongside species‐specific or trait‐specific phenological responses to climate change, interactions between different species and taxa at a range of trophic scales may also be impacted. Especially relevant to bees are plant–pollinator interactions. Temporal mismatches between wild bees and the plants on which they depend may arise for several reasons, each relating to differential impacts of climatic variability on two species (Stenseth & Mysterud, 2002). This can include interacting species responding (1) to different climatic cues (e.g. temperature vs. rainfall), (2) to the same climatic cue at different times (e.g. March temperature vs. May temperature) or (3) with different magnitudes to the same climatic cue during the same window. Loss of floral resources, such as pollen, has been proposed as one of the major drivers of wild bee decline (Biesmeijer et al., 2006; Scheper et al., 2014), and temporal mismatches have been shown to reduce flower visitation, thereby reducing floral resource availability. In turn, this could possibly impact offspring size—which may negatively impact offspring survival (Slominski & Burkle, 2021) and potentially longer term population persistence. Current evidence for phenological mismatches in plant–pollinator interactions is mixed, with the majority of interactions tracking each other temporally. There are, however, certain interacting partners, especially in early‐season interactions, that show independent shifts in phenology, such as seen between the flowering plant *Corydalis ambigua* and its bumblebee pollinators in relation to the timing of snowmelt in observations from northern Japan (Kudo & Ida, 2013).

Long‐term, ad hoc records of bee sightings may provide a useful proxy for phenology and have been used to generate estimates for emergence dates in numerous other studies (Brooks et al., 2014; Olsen et al., 2020). Ad hoc hoverfly recording schemes produce similar phenological estimates to those produced by standardized hoverfly recording schemes (Hassall et al., 2017), suggesting suitability for phenological studies. As with hoverflies, a long‐term database of bee records exists in Great Britain, collected and verified for accuracy by expert taxonomists of aculeate Hymenoptera, by the Bees, Wasps and Ants Recording Society (BWARS, www.bwars.com), which holds records dating back over 100 years.

Understanding the impact of temperature on phenology not only provides a picture of the past but may also allow for predictions of future flight dates, which in turn can help inform future conservation efforts through ‘phenological matching’ of bee flight and suitable forage plants (Russo et al., 2013). It is predicted that emergence dates of British bees will (1) be gradually becoming earlier in the year, and that (2) emergence dates are earlier in warmer years compared with cooler ones. It may also be the case that (3) specific life‐history traits determine sensitivity of emergence dates to temperature increases. Therefore, this study looks to make use of this long‐term data set to answer the following questions:

1. How have the emergence dates of British bee species changed over the past 40 years?
2. Does temperature play a role in any changes observed in emergence dates?
3. Do specific life‐history traits influence temporal shifts in bee emergence dates?

## 2 METHODS

### 2.1 Bee data

The BWARS of Great Britain and Ireland provided records of bee sightings ‐ although only records from Great Britain were used. This comprises an opportunistic, predominantly observational data set, where contributors can submit records containing a species, a sighting date and a location. While anyone could submit records to this database, to be eligible for inclusion in this data set records must meet a data quality threshold, where the data is checked and verified by experts within BWARS for taxonomic accuracy. BWARS coordinates a network of regional coordinators, an expert entomologist specialising in Hymenopteran species (Sumner et al., 2019). In cases where species identification is questionable, consultation between the observer and coordinator takes place, and if the record cannot be verified to the satisfaction of the coordinator, it is not included in the data set.

To ensure robust estimates of bee emergence dates, only species that met a minimum threshold of 20 years of data with 20 or more records per year were included in the analysis. This threshold was met by 88 species of bees. Records for these species were extracted for the period 1980–2019 (Table S1), as this period provides the most abundant data, for a total of 363,724 records. For univoltine species (71 species) the fifth percentile flight date for each species, in each year, was calculated and is hereafter referred to as the ‘emergence date’ and is 5% of the distance between the first and last record. For the 7 bivoltine and 10 species with variable voltinism, a *k*‐means clustering analysis was used to identify which generation each record belonged to, and only those records in the earlier generation were used in the fifth percentile estimation. This was done to reduce the influence of the second generation on the predicted emergence date.

Traits data were obtained from the European Bee Traits Database. Data for four traits were extracted for the 88 bee species and are listed in Table 1. These were the ‘Emergence period’, ‘Voltinism’, ‘Lecty’ and ‘Overwintering stage’. Species were then grouped into trait groups, with each group comprising species with the same characteristics across all four traits.

**TABLE 1:** Traits selected for phenological sensitivity analysis.

Trait	Levels
Emergence period	‘Spring’—Mean emergence (1980–2019) in March, April or May ‘Summer’—Mean emergence (1980–2019) in June, July or August
Voltinism	‘Univoltine’—Species has one generation per year Bivoltine—Species has two generations per year ‘Variable’—Species varies in the number of generations per year across the study period.
Lecty	‘Polylectic’—Visits a wide range of unrelated plant species for pollen ‘Oligolectic’—Visits a narrow range of plant species from a single plant family for pollen ‘Clepto‐ and social parasites’—Cleptoparasites and social parasites
Overwintering stage	‘Adult (female only)’—Females overwinter as adults ‘Adult within nest’—Overwinter as adults within cocoon ‘Prepupa’—Overwinters as prepupa

### 2.2 Climate data

The mean daily temperature from 1979 to 2019 was obtained at a 0.25° gridded resolution from the ensemble mean of the E‐Obs data set v26.0e (Cornes et al., 2018), and the mean value of all grid squares covering Great Britain was extracted.

A temperature record was then assigned to each phenophase record. The timing of this window varied on a species‐by‐species basis. For each species, first, the mean emergence date across all years was calculated. This was termed the ‘reference date’. The climate window ran from the 90 days leading up to and including the reference dates. For example, for a species with a mean emergence date across all years of 17 April, the temperature window would begin to run on 18 January (17th in leap years), and end on 17 April.

### 2.3 Statistical analysis

#### 2.3.1 Phenological shift over time

A two‐step process, similar to that employed by Bartomeus et al. (2011), was used to estimate phenological trends over time. This included looking at community (all 88 species considered as a group) and species (all 88 species considered individually) level trends. First, the shift in emergence dates of all bee species over time was tested using a linear mixed model regressing emergence date as a function of year, with the number of records making up each estimate (*n*) and the mean northing of each emergence estimate (*northing*) also included as fixed effects, to take into account issues related to sampling effort and sampling distribution. Species was included as a random effect. Mixed models were run using the package ‘nlme’ (Pinheiro et al., 2017), and marginal and conditional *R* ^2^ were calculated using the ‘performance’ package (Lüdecke et al., 2021).

Subsequently, the data set was split into individual species, and species‐level linear models were run regressing emergence date against year, while again accounting *n* and *northing* to estimate the shift in emergence dates over time for each species. The estimate of these models was taken to be the temporal shift in emergence dates (days per year). These models were run with a Benjamini–Hochberg correction for multiple tests to avoid Type I errors (*q* = 0.05) (Benjamini & Hochberg, 1995).

#### 2.3.2 Phenological sensitivity to climate change

A similar approach was used to estimate the sensitivity of bee emergence dates to temperature change. First, a linear mixed model was run with emergence date as a function of mean temperature during the 90 day window, *n* and *northing* as fixed effects, and again accounting for variation between species by including it as a random factor. The estimate was once again taken to be the sensitivity of each phenophase to increasing temperature (days per °C). Second, species‐level linear models were run with the regressing emergence date against mean temperature for the 90 days prior to the reference date, including again *n* and *northing* in order to estimate the shift in emergence dates in relation to temperature for each species. Once again, species‐level models were run with a Benjamini–Hochberg correction for multiple tests to avoid Type I errors (*q* = 0.05).

#### 2.3.3 Impact of traits on phenological sensitivity to climate change

The Kruskal–Wallis test (Kruskal & Wallis, 1952) was used to test for differences in phenological sensitivity of emergence dates to temperature change between ‘trait groups’ that differed by only one trait. Trait groups are defined as species which share all four traits (Table 2). This helps to overcome the fact that many traits often overlap (e.g. all species that overwinter as adults within a cocoon are also spring emerging species). By comparing trait groups that differ by only one trait, it isolates the effects of an individual trait on sensitivity of emergence dates to temperature change (Dorian et al., 2022). Phenological sensitivity of emergence dates to temperature change was taken as the species‐level estimates from the linear models described in the previous section. Where the Kruskal–Wallis test indicated significant differences between groups, the Dunn test (Dunn, 1964) was used to identify which pairs significantly differ from each other, again using a Benjamini–Hochberg correction for multiple comparisons (*q* = 0.05). This test was carried out using the package ‘FSA’ (Ogle & Ogle, 2017).

**TABLE 2:** Trait groupings and number of species within each trait group.

Trait group	Trait				Species
	Lecty	Overwintering stage	Emergence period	Voltinism	
**A**	**Polylectic**	**Adult within cocoon**	**Spring**	**Univoltine**	**15**
**B**	**Polylectic**	**Adult within cocoon**	**Spring**	**Bivoltine**	**5**
**C**	**Polylectic**	**Adult within cocoon**	**Spring**	**Variable**	**4**
**D**	**Oligolectic**	**Adult within cocoon**	**Spring**	**Univoltine**	**3**
E	Polylectic	Prepupa	Summer	Univoltine	10
F	Oligolectic	Prepupa	Spring	Univoltine	1
G	Polylectic	Prepupa	Spring	Univoltine	1
H	Oligolectic	Prepupa	Summer	Univoltine	2
I	Oligolectic	Adult within cocoon	Spring	Variable	1
J	Clepto‐ and social parasite	Adult (female only)	Spring	Univoltine	12
K	Polylectic	Adult (female only)	Summer	Univoltine	1
**L**	**Polylectic**	**Adult (female only)**	**Spring**	**Univoltine**	**22**
M	Polylectic	Adult (female only)	Spring	Multivoltine	2
N	Clepto‐ and social parasite	Prepupa	Summer	Univoltine	2
O	Polylectic	Prepupa	Summer	Variable	4
P	Polylectic	Adult (female only)	Spring	Variable	1
Q	Oligolectic	Adult within cocoon	Summer	Univoltine	2

*Note*: Groups in bold were used to test for differences in phenological sensitivity of emergence dates to climate change.

Only trait groups containing a minimum of three species were used in this analysis, resulting in a total of eight trait groups available for comparison. Groups A (univoltine), B (bivoltine) and C (variable) were compared, which differ only in voltinism. These groups share the same traits for the other three trait categories, all being polylectic, spring emerging species which overwinter as adults within a cocoon. Second, Groups A (polylectic) and D (oligolectic) were compared, which differed only in lecty, with both groups containing spring emerging, univoltine species which overwinter as adults within a cocoon. Finally, Groups A (adult within cocoon) and L (adult—female only) were compared, these groups differ only in overwintering stage, containing species which shared the other three traits—containing spring emerging, univoltine, polylectic species.

Additionally, to test for a taxonomic trend, a Kruskal–Wallis test and subsequent Dunn test with Benjamini–Hochberg correction for multiple comparisons were run to compare sensitivity of emergence dates to temperature change at the genus level. Again, only genera containing 3 or more species were included, allowing for comparisons between seven genera (*Andrena*, *Bombus*, *Hylaeus*, *Lasioglossum*, *Megachile*, *Osmia* and *Sphecodes*).

## 3 RESULTS

### 3.1 How have the emergence dates of British bee species changed over the past 40 years?

**FIGURE 1:**
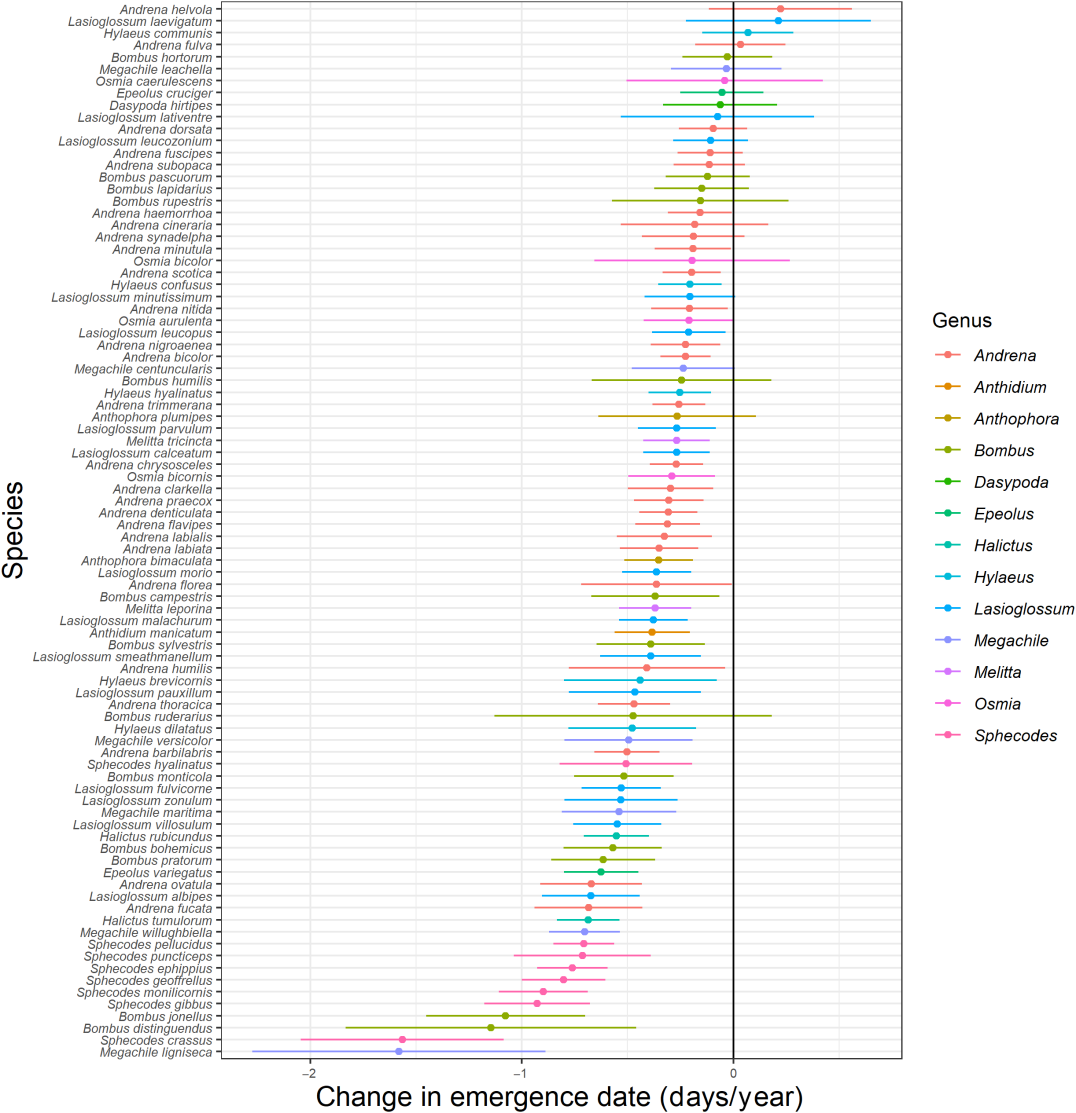
Change in species' emergence dates per year. Error bars indicate standard error.

When considered as a group, emergence dates showed a significant advance throughout the study period (0.40 ± 0.02 days per year, *p* < .001). There was also a significant effect of *n* on emergence phenology, with increasing records also linked to earlier emergence estimates (0.005 ± 0.002 days per additional record, *p* < .018). *Northing* did not have a significant effect in the community time model (*p* = .972). The fixed effects explained relatively little of the variation in this model (marginal *R* ^2^ = .018, conditional *R* ^2^ = .857). At the species level, 14 species (15.9%) showed a significant advance in emergence dates over time, ranging from 0.50 ± 0.15 days per year (*Andrena barbilabris*, *p* = .021) to 1.56 ± 0.48 days per year (*Sphecodes crassus*, *p* = .028) (Figure 1). *R* ^2^ values from the species‐level models ranged from .009 to .485, with a mean of .231. Full model results are available in Table S2 and species' level plots in Figure S1.

### 3.2 Does temperature play a role in any changes observed in emergence dates?

**FIGURE 2:**
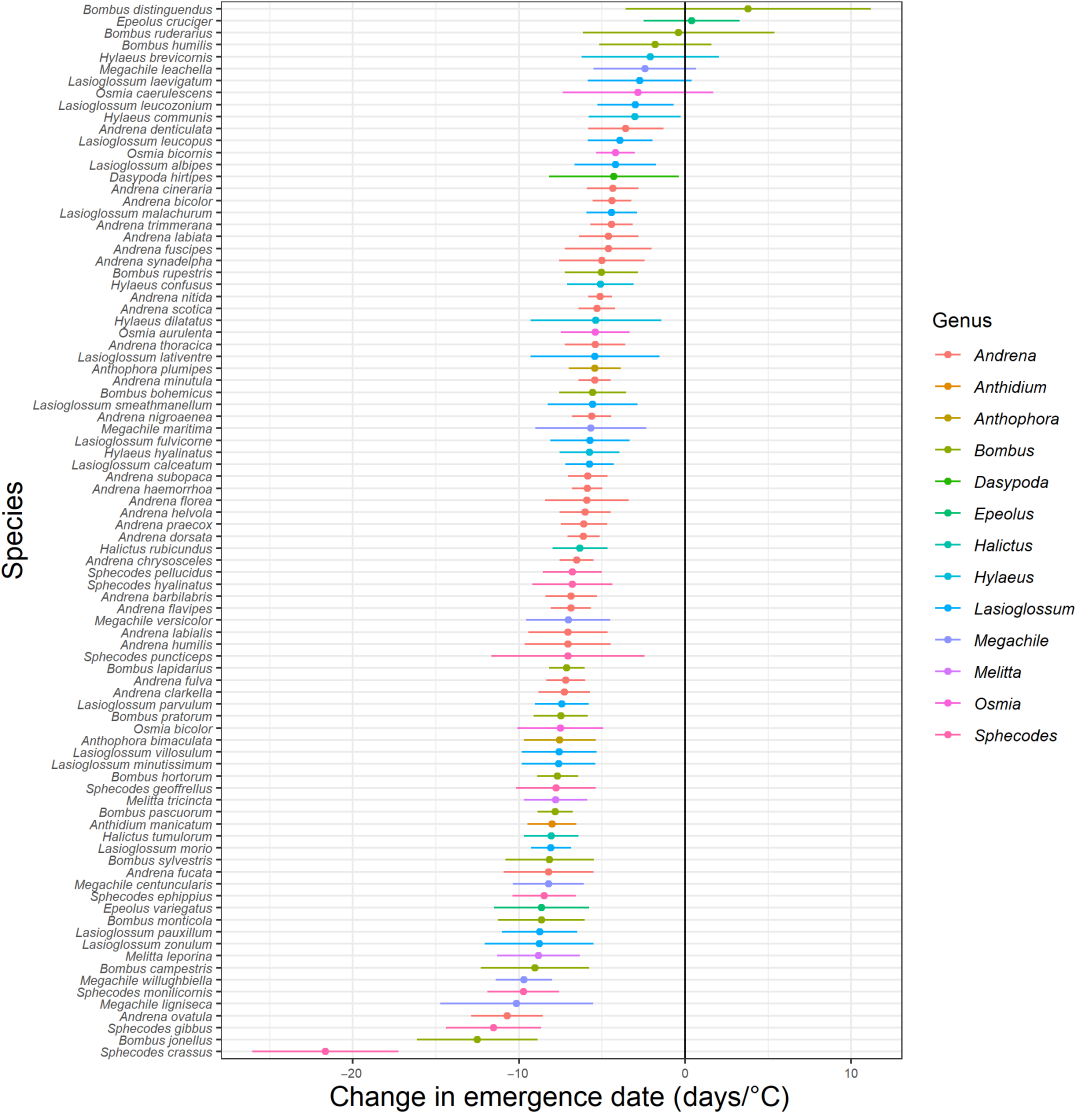
Change in species' emergence dates per °C temperature increase. Error bars indicate standard error.

Emergence dates were significantly earlier in years with warmer average temperatures (90 days preceding mean emergence date), at a rate of 6.5 ± 0.2 days per 1°C temperature increase (*p* < .001) across all species as a group. Neither *n* (*p* = .139) nor *northing* (*p* = .402) had a significant effect on emergence dates in the community temperature model. Again, the fixed effects accounted for relatively little of the variation in this model (marginal *R* ^2^ = .092, conditional *R* ^2^ = .940). There was variation in individual species' responses to temperature change, with emergence dates of 67 species (76.1%) showing a significant advancement in warmer years. Sensitivity ranged from a 4.2 ± 1.2‐day advance in emergence date per °C temperature increase (*O. bicornis*, *p* = .029) to a 21.7 ± 4.4‐day advance in emergence date per °C temperature increase (*S. crassus*, *p* = .029) (Figure 2). *R* ^2^ values of species level models ranged from .029 to .722, with a mean of .371. Full model results are available in Table S3, and species' level plots in Figure S2.

### 3.3 Do specific traits influence temporal shifts in bee emergence dates?

Separate Kruskal–Wallis tests were conducted to assess whether there were significant differences in the median values of phenological sensitivity of emergence dates to temperature warming (estimates of linear models calculated in previous section) between three sets of groups, each of which differ by a single trait (A, B and C—voltinism, A and D—lecty, A and L—overwintering stage) and between different genera.

**FIGURE 3:**
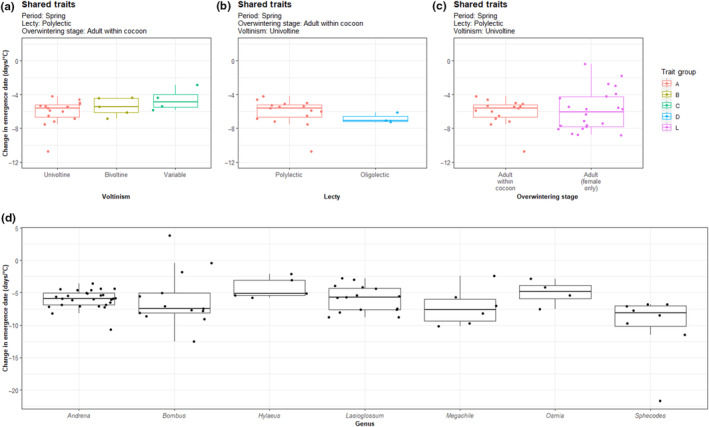
Comparison of sensitivity of emergence dates to temperature increase of trait groups sharing three traits and differing by (a) voltinism, (b) lecty and (c) overwintering stage, and comparison of sensitivity of emergence dates to temperature increase of different genera (d).

None of these comparisons yielded significant differences in phenological sensitivity of emergence dates to temperature warming. This included the comparison between different levels of voltinism in spring emerging, polylectic species, which overwinter as adults within a cocoon (*χ* ^2^ = 2.63, df = 2, *p* = .269, Figure 3a). There was also no significant difference in sensitivity of emergence dates to temperature change between oligolectic and polylectic species in spring emerging, univoltine species, which overwinter as adults within a cocoon (*χ* ^2^ = 2.19, df = 1, *p* = .139, Figure 3b). Finally, no significant difference in sensitivity of emergence dates between different overwintering stages in spring emerging, univoltine, polylectic species (*χ* ^2^ = 0.16, df = 1, *p* = .688, Figure 3c).

For the taxonomic comparisons, the Kruskal–Wallis test revealed significant differences between at least one pair of genera (*χ* ^2^ = 15.26, df = 6, *p* = .018, Figure 3d). The subsequent Dunn test revealed emergence dates of only two pairs of genera had significantly different sensitivities to temperature change, *Hylaeus* and *Sphecodes* (*p* = .022), and *Andrena* and *Sphecodes* (*p* = .045).

## 4 DISCUSSION

This study utilises 40 years of British bee records for 88 species to present evidence of climate‐driven temporal shifts in the phenology of a wide range of British bees. When all species are considered as a group, the mean emergence date has advanced on an average by 0.40 ± 0.02 days per year, with species‐level linear models revealing significant interspecies variation in this advance. The scale of the advancements here are over twice as severe as similar studies from different parts of the globe, with Bartomeus et al. (2011) reporting a 0.18 ± 0.05 days per year advance in bee phenology in the US between 1970 and 2010 and with Dorian et al. (2022), who reported a shift of 0.16 ± 0.06 days per year between 1970 and 2022, also in the US.

We also add to the growing body of evidence that phenological shifts are linked to climate, in this case, temperature, with an average advance of 6.5 ± 0.2 days per 1°C rise in temperature, across all species as a group. Again, these advances were species‐specific, with individual advances of up to 21.7 ± 4.4 days per 1°C rise in temperature (*Sphecodes geofrellus*). This advance is also more severe than the findings of Bartomeus et al. (2011), who found the average collection day of museum specimens advanced by 3.6 ± 0.2 days per 1°C temperature increase in mean April temperatures. However, the findings of both this study and Bartomeus et al. (2011) point to changing climatic conditions being a major driver of bee phenology, while also indicating potential regional differences in phenological responses to temperature. The differences in findings could also be due, in part, to differences in bee communities, none of the species studied by Bartomeus et al. (2011) were used in this study.

This study also adds to the growing body of evidence that phenological shifts are not uniform and vary between species, although shifts in emergence dates do not appear to be driven to any great degree by specific life history traits. Pairwise comparisons of changes in emergence dates of different groups of species that differed by only one trait showed no significant difference between any pairs. This finding is contrary to results from Dorian et al. (2022), who found differences in phenological sensitivity between species with different activity periods and nesting preferences, a trait not tested here due to lack of variation in nesting preference between species.

While this study was not able to isolate a specific trait that impacts sensitivity of emergence dates to temperature, not all traits were tested. Other traits, such as sociality or body size may impact sensitivity of emergence dates to temperature change. Sociality may impact the emergence estimates themselves, most of the *Bombus* species in this analysis are primitively eusocial, and the queens emerge before workers and males. Evidence for sociality as an important trait in determining phenological sensitivity to climate change is limited, with a non‐significant difference in rates of phenological change found between eusocial and solitary species (Bartomeus et al., 2011). Additionally, while this study did not explicitly account for a phylogenetic signal, comparing sensitivity of emergence dates to temperature by genera showed limited differences, with only two pairs of genera showing any significant differences (*Halictus* and *Sphecodes* and *Andrena* and *Sphecodes*). Again, this confirms findings by Bartomeus et al. (2011) that most of the variability in phenological shifts are at the species level rather than at higher taxonomic ranks.

The earlier emergence of bees highlighted here is likely to come with a range of consequences. For example, despite the warming climate, incidences of late‐spring frosts are increasing in Europe (Lamichhane, 2021; Zohner et al., 2020). While the direct impact of late frost on bees is somewhat buffered by their ability to insulate themselves from cold temperatures in their nests, especially for ground‐nesters, the plants they forage on do not have such ability and are at greater risk of damage. This may impact their attractiveness to bees by reducing the number of flowers and flower size (Pardee et al., 2018) or through a reduction or alteration the chemical composition of the rewards they offer (Akšić et al., 2015). Some plant groups have also been shown to receive fewer visits by pollinators after experiencing frost damage (Pardee et al., 2018). Although bees spent longer working frost‐damaged flowers compared with undamaged flowers, this could be because they are having to work harder to extract rewards, reducing their net energy gain. There is evidence that these plants are indeed undergoing shifts in flowering dates which could potentially expose them to this late frost risk (Büntgen et al., 2022; Fitter & Fitter, 2002).

Recent estimates suggest that plants in the UK are advancing first flowering dates by an average of 5.4 days per decade (Büntgen et al., 2022). Although there is significant species‐level variation in these shifts in plant phenology, plant phenological shifts are generally more pronounced than the average of a 4.0 day per decade advance in bee emergence reported in this study. Even seemingly small phenological mismatches between bee emergence and plant flowering can have severe implications for bee survival (Schenk et al., 2018; Slominski & Burkle, 2021). The evidence we present here, coupled with evidence of shifts in plant phenology, highlight the potential for phenological mismatches.

While we show that climate plays a role in determining bee phenology, it is probable that temperatures over a fixed 90 day window are not the most biologically meaningful predictor of emergence dates at the species level (Bailey & van de Pol, 2016). Bee emergence can likely be better predicted using a species‐specific time window, through the implementation of a sliding window or climate window analysis (Bailey & van de Pol, 2016). Future work is therefore recommended to refine the exploratory models presented here to find more biologically meaningful climate windows for explaining phenological trends of species of interest. Additionally, exploration of other potential climatic factors and extreme events that may influence bee emergence dates, such as rainfall, drought or frost may also be an important area of future research. This is recommended as understanding which climatic factors and time windows are good predictors of emergence, in conjunction with climate projections could enable predictions of future emergence dates. In turn, this could allow for better‐timed conservation interventions (Russo et al., 2013).

While more work is required to refine the temporal windows of the models presented here, it provides a framework for utilising long‐term citizen science data to assess phenological shifts in British bees. To conclude, analysis of this long‐term data set reveals that many British bee species are advancing their emergence dates, and that these advances are likely to continue with further climate warming. Comparison with similar analyses on flowering plants suggests that bee emergence is less sensitive to climate change than flowering dates, highlighting a potential risk of phenological mismatch, which could lead to major disruption of vital pollination networks. Finally, we recommend investigating the development of more refined models to better predict bee emergence dates to further our understanding of climate‐induced shifts in bee emergence to evaluate potential risks of future phenological mismatches.

## AUTHOR CONTRIBUTIONS

**Chris Wyver:** Conceptualization (lead); investigation (lead); methodology (lead); visualization (lead); writing – original draft (lead); writing – review and editing (equal). **Simon G. Potts:** Conceptualization (supporting); formal analysis (supporting); methodology (supporting); supervision (supporting); writing – review and editing (equal). **Mike Edwards:** Data curation (equal); writing – review and editing (equal). **Rowan Edwards:** Data curation (equal); writing – review and editing (equal). **Stuart Roberts:** Data curation (equal); writing – review and editing (equal). **Deepa Senapathi:** Conceptualization (supporting); formal analysis (supporting); investigation (supporting); methodology (supporting); supervision (lead); writing – review and editing (equal).

## FUNDING INFORMATION

This project was funded by BBSRC (Grant number: BB/T508895/1) and Waitrose Agronomy Group as part of the Waitrose Collaborative Training Partnership. WorldWide Fruit Ltd contributed to the funding and development of this project.

## CONFLICT OF INTEREST STATEMENT

The authors declare no conflict of interest.

## Supporting information


Figures S1–S2

Tables S1–S3

## Data Availability

The data that support the findings of this study are openly available in the University of Reading Data Repository at https://doi.org/10.17864/1947.000477.
