# Supplementary material for: Climate‐driven phenological shifts in emergence dates of British bees
Source: Ecol Evol. 2023 Jul 9;13(7):e10284. doi: 10.1002/ece3.10284 (PMC10329875; doi:10.1002/ece3.10284)
Supplement: Supplementary file 1 — Figures S1–S2 [file ECE3-13-e10284-s002.docx]

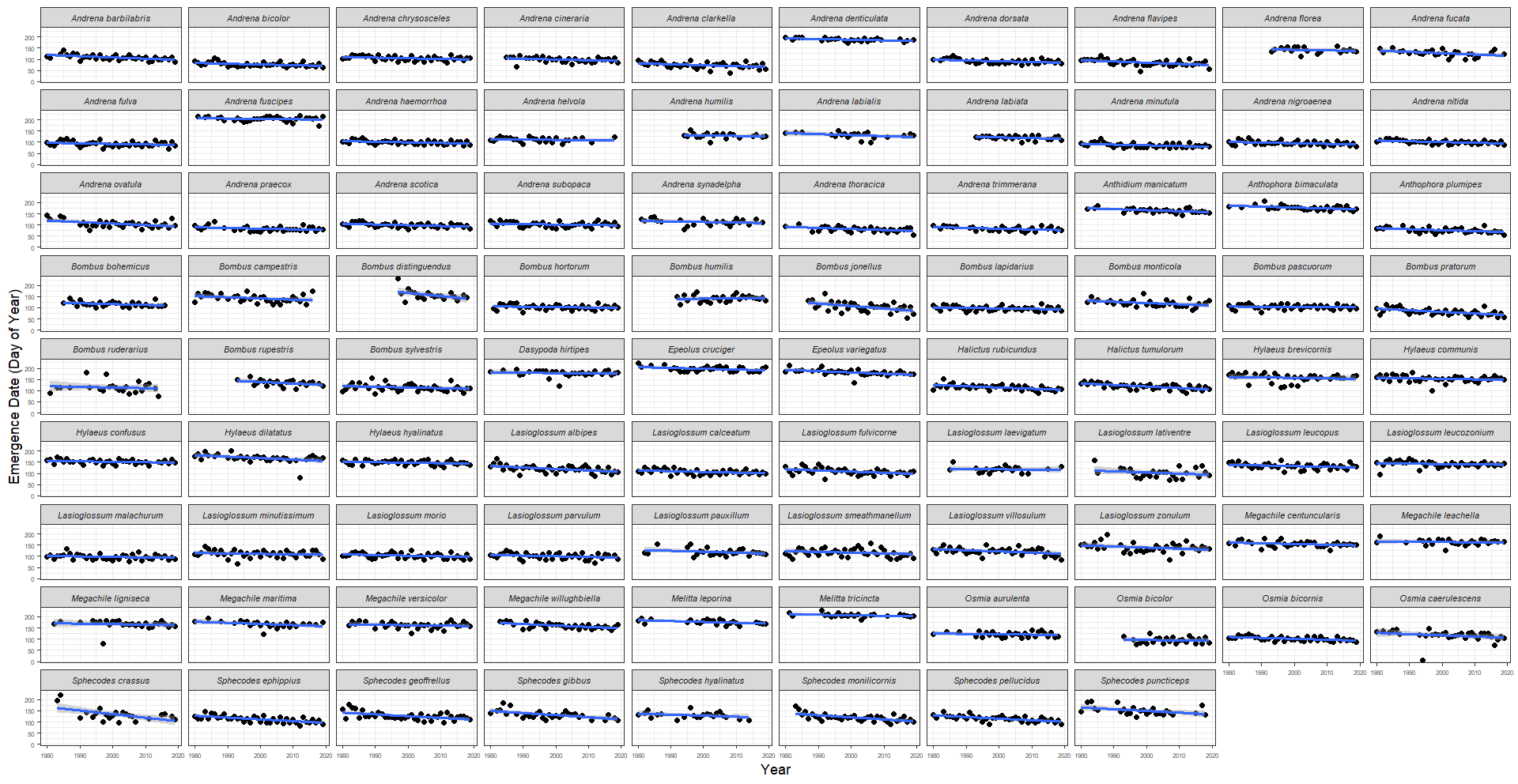


**Figure S1.** Trends in bee emergence dates over time. Shaded area represents 95% confidence intervals.


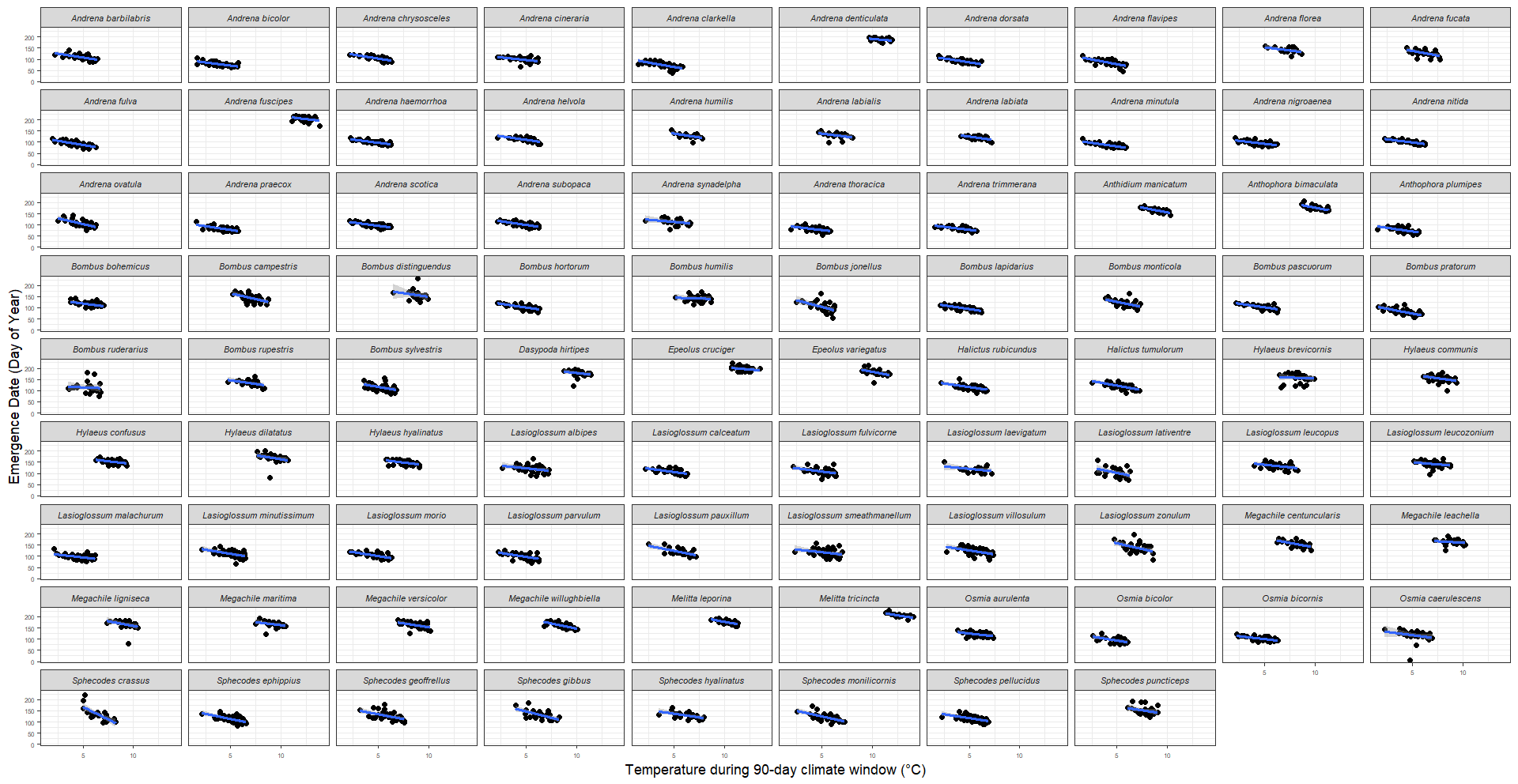


**Figure S2.** Trends in bee emergence dates against temperature. Shaded area represents 95% confidence intervals.
